# Suicide in Healthcare Workers: Determinants, Challenges, and the Impact of COVID-19

**DOI:** 10.3389/fpsyt.2021.792925

**Published:** 2022-02-03

**Authors:** Sana Awan, Mufaddal Najmuddin Diwan, Alifiya Aamir, Zoha Allahuddin, Muhammad Irfan, Alessandro Carano, Federica Vellante, Antonio Ventriglio, Michele Fornaro, Alessandro Valchera, Mauro Pettorruso, Giovanni Martinotti, Massimo Di Giannantonio, Irfan Ullah, Domenico De Berardis

**Affiliations:** ^1^Department of Internal Medicine, Dow Medical College, Karachi, Pakistan; ^2^Department of Internal Medicine, Hayatabad Medical Complex, Peshawar, Pakistan; ^3^Department of Mental Health, Azienda Sanitaria Unica Regionale 5 Marche, San Benedetto Del Tronto, Italy; ^4^Department of Mental Health, Mental Health Center of Ortona, Azienda Sanitaria Locale Chieti, Chieti, Italy; ^5^Department of Mental Health, University of Foggia, Foggia, Italy; ^6^Laboratory of Molecular and Translational Psychiatry, Unit of Treatment-Resistant Psychosis, Section of Psychiatry, University of Naples Federico II, Naples, Italy; ^7^Department of Neuroscience, Imaging and Clinical Sciences, University “G. d'Annunzio” of Chieti - Pescara, Chieti, Italy; ^8^Department of Internal Medicine, Kabir Medical College, Gandhara University, Peshawar, Pakistan; ^9^Department of Mental Health, Psychiatric Service for Diagnosis and Treatment, Hospital “G. Mazzini”, Teramo, Italy

**Keywords:** COVID-19, healthcare workers, suicide, depression, anxiety, burnout, post-traumatic stress, substance abuse

## Abstract

The Coronavirus disease-19 (COVID-19), which first appeared in Wuhan, China, and was later declared a pandemic, has caused significant morbidity and mortality worldwide. Numerous efforts have been made worldwide to understand the disease's physical manifestation. However, less emphasis has been placed on the pandemic's mental health challenges for healthcare workers (HCWs) who played a critical role in fighting the disease. Existing literature shows the detrimental psychological impact and increased incidence of depression and anxiety among HCWs. It is expected that the mental health crisis will become a serious issue affecting HCWs, with long-term negative consequences following COVID. Physicians and nurses already represent the highest risk groups of suicide among the general population, and suicide can be regarded as an occupational hazard in the healthcare industry. Increased workload, burnout and fatigue, multifaceted challenges women HCWs, and increased substance abuse are contributing factors to suicide ideation. In this article, we identify the risk factors of suicide among HCWs, discuss mental health challenges exacerbated by the pandemic and its impact on suicide ideation.

## Introduction

The Coronavirus disease 2019 (COVID-19) pandemic has completely transformed the lives of millions of people worldwide, putting enormous strain on society and healthcare systems. The disease has now affected 213,237,126 individuals, resulting in 4,452,903 deaths (1). The new reality of lockdown, self-isolation, fear, and uncertainty surrounding this disease has significantly harmed the mental health of many. During the pandemic, about 4 in 10 adults in the U.S. reported anxiety or depressive disorder symptoms (2). Similarly, a nationwide survey in China reported that 35% of the respondents had experienced psychological distress due to the SARS-CoV-2 (3). The 2021 World Health Organization (WHO) annual health report recorded 7,00,000 deaths by suicide globally (4). Latest reports show that it is currently the 17th leading cause of death (5).

The trend of suicide occurrence observed globally does not show association with a particular age group; however, the vulnerability to suicide increases with age and puts older adults at the most significant risk (4). Moreover, despite being described as a “global phenomenon” by the WHO, 777% of the global burden of suicide stems from low-and middle-income countries (LMICs). Prevalent poverty and low socio-economic standing rapidly increased suicidal ideation amongst populations residing in LMICs (6).

Despite a disproportionate burden, the crude death rate is highest in the European region, with 12.8 deaths per 1,00,000 population (4). In the United States, a steady rise of 30% has been observed in the number of deaths by suicide during 2000–2016. In 2017, this rate increased to 14.0 per 1,00,000. The WHO annual statistics also show an apparent gender predisposition with crude death rates in men nearly twice women (4).

Recent reports from 2020 show that mental health amongst physicians and frontline workers has been affected by the current COVID-19 pandemic (7, 8). Despite the risk of infection and death, HCWs have been at the frontline in the global fight against the pandemic. Fear of exposure to infection and transmission, staff shortages, inadequate personal protective equipment, and work stress has added an extra burden to an already stressful lifestyle (8). All these factors eventually have led to a rapid progression to burnout and chronic fatigue, which can progress to post-traumatic stress disorder (PTSD). A systematic review assessing the mental health problems in HCWs since the pandemic reported that nurses, women workers, frontline health care workers, younger medical staff, and workers in areas with higher infection rates had faced the highest level of psychological distress (9).

As a result, it is critical to assess the repercussions of declining mental health, address and acknowledge the job strain, particularly for women, and identify high-risk individuals to implement early prevention initiatives. This article discusses the mental health issues that HCWs face and variation in suicide rates based on their age, gender, specialty, and working conditions. Furthermore, we address the underlying causes of burnout and emotional distress among HCWs due to the effects of the COVID-19 pandemic.

We believe that addressing these factors is critical in developing a comprehensive and targeted strategy for reducing the risk of Suicide among HCWs. Early prevention initiatives, identifying at-risk individuals, and focused treatment for those in need are all part of this strategy.

## Differentiating Characteristics in Suicide Rates

Different reports have been made available regarding suicide concerning age globally. According to a report from 2018, suicide has been reported to be the most common cause of death during the second and third decades of life (10). Previous reports suggested an overall increase in the tendency to commit suicide; however, the evaluation of recent data suggests an increase in rates of suicide in 33 countries alongside a decrease observed in 15 countries. All reports have been classified according to gender, and a significant increase has been observed in females from 37 countries and in males from 33 countries. The effect of other factors on this trend was also described (11). Factors including the socio-economic status, mortality rate, income inequality in females, the proportion of Gross Domestic Product (GDP), and the per capita expenditure on healthcare significantly impacted age-related trends in suicide.

A review from 2004 suggests that the mortality ratio due to suicide amongst gender was markedly higher for female physicians than male physicians. (12) A recent meta-analysis has confirmed that the suicide mortality rate in male physicians was considerably lower than the general population. However, assessment of rates of suicide deduces male sex as a significant risk factor. Suicide is historically known as an aggressive behavior not to be observed amongst females. A study from 2008 suggested that white males are more likely to commit suicide, whereas, in China, Suicide is seen as a cry for help and is more common amongst females (13). Overall, less importance has been given to female statistics of suicide, and the limited literature on physician suicide does not highlight important risk factors. Multiple reports also confirm that females have a higher chance of experiencing suicidal ideation. Higher suicide rates were also reported in countries experiencing political reform and social environment changes. This validates a concern that females are more affected since reform changes gender roles, increasing the chances of burnout among females (14).

According to multiple reports, the risk of committing suicide correlates to the vocation individual works in. A study analyzing the different demographics of cases of suicide shows that the risk ranged from 2.73 in physicians to 0.44 in architects and engineers (15). When assessing the risk of suicide, easier access to means of suicide is a significant risk factor. According to a report, it was confirmed that healthcare professionals, including nurses, doctors, and pharmacists, are more inclined to use poisons as a means of suicide (16).

Within healthcare itself, multiple specialties are specifically associated with a higher frequency of suicide. The majority of studies deduce the effect of the quality of work affecting mental health by reporting symptoms of PTSD and depression in these workers. An additional factor leading to physician suicide can be attributed to the development of chronic mental health disorders such as post-traumatic stress disorder, substance abuse, and depressive mood disorder. Mental illness has always been associated as a risk factor for suicide.

Emergency responders are on the front line of trauma and experience highly stressful work-related conditions. They have often been exposed to critical medical situations and regularly witnessed fatalities. According to a review, 21.4–53.7% of emergency responders from the Great East Japan earthquake and Lebanon-Israel War fit the criteria for depression (17). The prevalence of PTSD among such groups was estimated to be higher than predicted, exacerbated by certain risk factors. A responder's likelihood of developing PTSD includes having no social support, certain neurotic and distant personality types, and individuals with harmful coping mechanisms.

In healthcare, competitive specialties like general surgery, anesthesiology has a noticeably higher risk of suicide due to multiple factors such as access to drugs and a high-stress work environment, which increase the likelihood of suicide. A meta-analysis report by Dutheils et al. evaluating suicide amongst specialties deduced a higher incidence in internal medicine (16%) followed by psychiatry (11%) and anesthesiology (4%) (18).

## Work Associated Factors For Suicide

Since hospital working conditions vary between and within regions, differences are expected in healthcare workers' mental health and suicide rates. Burnout is a psychological condition that develops when people are exposed to a stressful work environment with high job demands and limited resources (19, 20). Suicidal ideation was found to be higher among those with burnout than those without it, according to a cross-sectional survey of 2,734 female nurses working in Taiwanese hospitals. Similarly, burnout was predictive of suicidal ideation in a longitudinal study of U.S. medical students, even when depressive symptoms were not present (21, 22).

To avoid the unanticipated effects of exhaustion and burnout, it is critical to strike a balance between appropriate working hours for health care workers, including surgical trainees, physician assistants, psychiatrists, and nurses, while maintaining high-quality patient care. A study conducted in Taiwan has shown that the prevalence of high work-related burnout from highest to lowest was nurses (66%), physician assistants (61.8%), physicians (38.6%), administrative staff (36.1%), and medical technicians (31.9%), respectively (23).

Residents, in particular, have been reported to be at a higher risk of depression as a result of a transition in their job capacity from a medical student to a trainee, which now includes intensive training hours along with studies and research (24, 25). This is compounded by characteristics such as early employment uncertainty, competitive work environment, and being younger, all of which are risk factors for emotional distress and ultimately suicidal thoughts. (26) reported that working in a training set for more than 80 h per week considerably increases the resident's depressive symptoms. Another study of first-year Japanese residents has found that 22.6 percent of residents had newly developed depressive symptoms after three months of residency (27).

Some places, particularly in the developed Western world, recognize and address the need to promote a better work environment by reducing working hours for doctors. The European Working Time Directive (EWTD) is one such initiative that has had a significant impact on training and work schedules in countries such as New Zealand and Australia. For example, an agreement in New Zealand specifies regular working hours for surgical trainees as 40 h per week and no more than 8 h per day, with overtime permitted and compensation (28). The impact of such reforms can be seen in a meta-analysis that identified a higher incidence of physician suicide in North America than in Australia, New Zealand, and the Pacific, and that suicide has declined over time, particularly in Europe (18).

It is important to note that job uncertainty and a shorter duration of employment are associated with a higher risk of suicide among healthcare professionals. This correlation was discovered among diagnostic medical radiation workers in South Korea, who had a 2.74 and 4.66 times greater risk of suicide among male and female workers with < one year of employment, respectively, than those with more than 10 years of employment (29). As a result, it appears that the healthcare workers with short-term jobs are a vulnerable population who should be given enough assistance and a better working environment (29). In addition, factors such as limited resources, an insufficient workforce, and high job expectations contribute to suicide risk among HCWs, especially in developing countries. This issue is more pronounced among nurses, one of the most undervalued groups, even though they are the backbone of every healthcare system. Nurses' suicide rates have gone unnoticed for years, and there are several underlying contributing factors, including long working hours, being the primary caregiver for patients, such as in the ICU, and a lack of professional autonomy (30).

Furthermore, female nurses who form the majority of the workforce have dual profession and home responsibilities. Davis et al. reported that the suicide incidence among nurses was 17.1 per 1,00,000, compared to 8.6 per 1,00,000 among women in the general population, representing a doubling of risk (31). In addition, the increased rate of substance abuse, such as antidepressants, opiates, and amphetamines, which have been discovered to be more commonly used as poisoning methods for suicide attempts among this demographic, is an important issue to highlight (31, 32).

## Mental Health Challenges Faced by Healthcare Workers

Literature regarding the mental well-being of healthcare workers was limited until 2016 (33); however, recent studies have started focusing on the challenges faced by healthcare workers. It has been noticed that constant amounts of stress negatively impact healthcare workers. Acute signs of anxiety, depression, and burnout have been noticed at all levels of training in the medical profession. According to a report from 2014, symptoms specific to depression have been noticed in medical students, whereas signs of burnout are highly probable amongst residents during their training (33). Seventy four percent of residents have reported signs of exhaustion in a study from 2015, thus highlighting the burnout epidemic in the medical community. It is described as a feeling of inefficacy, depersonalization coupled with signs of emotional exhaustion in the workplace (34). Another study has reported that constant exposure to intense and unpredictable working hours could lead to fatigue during training years, causing multiple mental health issues amongst health care workers (35).

Specific categories of physicians affected by PTSD include emergency physicians, medical personnel working in remote areas or warzones, physicians in training, those indirectly exposed to trauma, and malpractice litigation (36). The frequency of dealing with a higher burden of death in trauma care puts them at a higher risk for developing these symptoms. High-risk groups for suicide are often those with either a previous history of such instances or those with Post-traumatic stress disorder (PTSD). PTSD symptoms have been observed in 30% of the residents working in the emergency room, with symptoms increasing with training (37). A 4-factor model suggests association patterns between suicidal ideation and symptoms of PTSD, which include re-experiencing feelings, negative alterations in cognition and mood, and hyperarousal. A similar study has suggested a 7-factor model correlating PTSD and an increased risk for suicide (17). Exposure to critically ill patients also poses a higher risk of developing depression amongst residents (33). Similarly, nurses and staff from the emergency room have a higher risk of experiencing suicidal ideation due to the nature of cases they consult daily.

Work-related changes are believed to play a significant role in developing mental health issues in physicians. In a study conducted among intensive care unit staff, almost half reported symptoms consistent with a probable diagnosis of post-traumatic stress disorder, severe depression or anxiety, or problem drinking. In the previous two weeks, 13% of respondents reported having recurring thoughts of being better off dead or injuring themselves (38).

According to studies conducted in the United States, 10–15% of HCPs will misuse substances at some point in their careers, and prescription drug abuse and addiction rates are five times higher among physicians than in the general population benzodiazepine opioid abuse have exceptionally high rates (39). According to a 2010 inquiry into the Texas Board of Nursing, around a third of all disciplinary actions against nurses were related to drugs or alcohol (39). Many healthcare personnel uses these medicines to relieve stress, depression, and pain symptoms and improve their general work performance. However, these seemingly harmless medications frequently become a source of dependence or addiction in the user over time.

## Impact of the Covid-19 Pandemic on Healthcare Workers' Mental Health

As the world and healthcare institutions deal with the fallout from the 2019 Coronavirus pandemic, we are likely to see the impact on the mental health of all healthcare professionals who are facing the reality of constrained resources and unthinkable choices, working to exhaustion, and caring for patients while being at significant personal risk. Fear and uncertainty about the new virus, its devastating repercussions, exhausting working hours in the hospital, countless deaths, and shortage of PPE's have all been significant setbacks for the healthcare system and have led to emotional stress and depressive symptoms among HCWs during the early stages of the pandemic (40). Coping with the COVID-19 pandemic has exacerbated HCWS's already-existing mental health issues and has placed extra strains on their well-being. Inevitably, this is bound to have a negative impact on the mental health of healthcare personnel, with long-term consequences.

Previous research has also shown that healthcare workers' mental health consequences of an epidemic or pandemic is long-term. Healthcare workers reported extreme emotional and traumatic stress and burnout, anxiety, and depressive symptoms during the SARS pandemic in 2003 and the MERS outbreak in 2015 (41, 42). Due to the nature of their job during the pandemic, HCWs, including nurses, emergency department staff, intensivists, and physicians, are among the most vulnerable groups to experience stressful events that can trigger a suicidal crisis (9). These individuals are already at risk for mental health issues such as post-traumatic stress disorder (PTSD), common among emergency room residents, intensivists, and surgeons (43).

According to studies, the COVID-19 epidemic has significantly increased stress, anxiety, depression, and insomnia among HCWs. The lack of standard treatment protocols or vaccines, work-related stress, the rapid spread of the virus, and fear of COVID-19 infection and transmission are major contributing factors leading to mental health disorders (44, 45). According to a study conducted among Malaysian HCWs, clinical depression has been the most significant predictor of present suicidal ideation, followed by mild (subthreshold) depression (46). Furthermore, it should be highlighted that female HCWs have appeared to be at a higher risk of suicidal behavior than their male counterparts during the current pandemic (47).

It concerns that a total of 26 worldwide COVID-19-related suicide cases among HCWs have been reported in a recent study; the affected persons consisted primarily of doctors, nurses, and paramedics, with more than half of them being female from India (45). In a similar report, multiple COVID-19-related suicides have been reported among nurses who have been on the frontlines caring for COVID-19 patients (48).

This emphasizes the importance of creating a supportive workplace environment for HCWs to maintain their mental well-being. It is critical to create less stressful schedules for HCWs in order to give them time to process and recover from their experiences, to recognize their vital contributions during this intense medical crisis, to remove the stigma around seeking help, and to provide timely and free psychological support to all HCWs.

## Recommendations For Suicide Prevention

It comes as no surprise that physicians have the highest suicide rate among all professions and that burnout rates for healthcare workers show an undeniable trend that the majority, if not all, face extreme levels of stress and burnout (49). Paradoxically, doctors are the least likely to seek mental health treatment. Furthermore, gender disparities are common, especially for women, when seeking care for psychological diseases and disclosing challenges like substance abuse. Men are more likely to seek specialized mental health treatment and primary inpatient care users (50). According to a survey, nearly half of female physicians who believed they fulfilled the criteria for a mental condition did not seek treatment (51). A tailored approach is required to break the stigma of seeking treatment based on gender stereotypes. While designing policies to remove barriers associated with mental health services, women healthcare workers' concerns about confidentiality and professional implications, such as effects on licensure status, should be considered. Hospital leadership and the medical community must develop and implement mental health and well-being strategies such as support groups, access to mental health professionals, and manageable schedules, as well as practicable workloads that will not only have a profound impact on the well-being of the physicians but will also help them provide higher quality care to their patients (44).

The Australian Medical Association's 'National Code of Practice – Hours of Work, Shiftwork and Rostering for Hospital Doctors' has been instrumental in changing attitudes to the ethics of safe hours and has decreased the proportion of doctors at high risk of fatigue (52).

Institutions and healthcare departments worldwide must develop comprehensive suicide prevention and mental health promotion plans in the future. Some integral components can include support and wellness programs for all healthcare workers, lifestyle and mental health screenings, and appropriate referrals for support or treatment. Ideally, at-risk behaviors should be identified via mental health screening, and interventions should be taken as early as possible by providing easier access to and communication with the mental health network. One example of such a suicide prevention initiative is the Healer Education Assessment and Referral (HEAR) program which provides education about risk factors and proactive screening focused on identifying, supporting, and referring clinicians for untreated depression and/or suicide risk (53).

Another concern that is necessary to address in the context of physician mental health and suicide risk is the reluctance of healthcare professionals in seeking help due to the risk of losing their medical license and higher medical liability insurance. Additionally, substance use is prevalent among HCWs, likely due to easy access to drugs (54). According to the Journal of Clinical Nursing, ~20% of all nurses struggle with an addiction to drugs or alcohol (55). It has been noted that they are more likely to commit suicide from poisoning and substance abuse than other suicide methods (56). Hence, the responsibility falls upon the organizations to provide a safety net for the physicians to seek treatment for drug addiction while addressing license and disciplinary issues in the workplace.

## Conclusions

It can be challenging to look beyond the immediate crisis during an emergency, but given the pandemic's long-term implications, we urge the healthcare community to address HCWs' mental health needs now and in the future. The ongoing pandemic stress-inducing factors are expected to exacerbate mental health concerns, including the increased risk of suicide.

As a result, it is critical to design and implement strategies that best support HCWs during crisis events; these interventions must be pragmatic, dynamic, and responsive according to individual needs. In addition, early prevention strategies, identification of at-risk individuals, and supportive workplace culture are essential to tackle the increased incidence of suicide among healthcare professionals.

## Author Contributions

All authors have contributed to the present review with equal efforts.

## Conflict of Interest

The authors declare that the research was conducted in the absence of any commercial or financial relationships that could be construed as a potential conflict of interest.

## Publisher's Note

All claims expressed in this article are solely those of the authors and do not necessarily represent those of their affiliated organizations, or those of the publisher, the editors and the reviewers. Any product that may be evaluated in this article, or claim that may be made by its manufacturer, is not guaranteed or endorsed by the publisher.
